# Real-World Activity and Safety of Metronomic Cyclophosphamide in Advanced Soft-Tissue Sarcoma: The Multicenter MeRCY Study

**DOI:** 10.3390/cancers18213497

**Published:** 2026-10-30

**Authors:** Clément Bolognini, Antoine Falcoz, Pauline Simon, Fabien Calcagno, Alice Hervieu, Nicolas Isambert, Isabelle Desmoulins, Sylvain Causeret, David Thibouw, Elsa Kalbacher, Guillaume Meynard, Dewi Vernerey, Loïc Chaigneau

**Affiliations:** 1 Department of Medical Oncology, Besançon University Hospital (CHU Jean Minjoz), 25000 Besançon, France; fcalcagno@chu-besancon.fr (F.C.); elsa.kalbacher@clermont.unicancer.fr (E.K.); lchaigneau@chu-besancon.fr (L.C.); 2 Université Marie et Louis Pasteur, EFS, INSERM, UMR RIGHT, 25000 Besançon, France; afalcoz@chu-besancon.fr (A.F.); dvernerey@chu-besancon.fr (D.V.); 3 Methodology and Quality of Life in Oncology Unit, Besançon University Hospital, 25000 Besançon, France; 4 Department of Pediatric Oncology and Hematology, Besançon University Hospital, 25000 Besançon, France; p1simon@chu-besancon.fr; 5 Département D’oncologie Médicale, Chirurgie et Radiothérapie, Centre Georges-François Leclerc, Unicancer, Université Bourgogne Europe, 21000 Dijon, France; ahervieu@cgfl.fr (A.H.); idesmoulins@cgfl.fr (I.D.); scauseret@cgfl.fr (S.C.); dthibouw@cgfl.fr (D.T.); 6 Department of Medical Oncology, Poitiers University Hospital, 86000 Poitiers, France; nicolas.isambert@chu-poitiers.fr

**Keywords:** soft-tissue sarcoma, metronomic chemotherapy, cyclophosphamide, geriatric oncology, real-world evidence, retrospective study

## Abstract

**Background/Objectives:** Metronomic cyclophosphamide (MCP) is sometimes used for advanced soft-tissue sarcoma (STS) when standard therapies are unsuitable. We assessed its activity and safety in routine practice. **Methods:** This French three-center retrospective study included 60 adults with unresectable or metastatic STS who started MCP in 2008–2021. Patients received oral cyclophosphamide 50 mg twice daily on days 1–7 of 14-day cycles; 12 (20%) also received prednisolone during treatment weeks. The primary endpoint was progression-free survival (PFS). **Results:** Median age was 70 years; 55% had ECOG performance status 2–3. Median PFS and overall survival (OS) were 2.6 months (95% CI, 1.8–3.6) and 6.4 months (95% CI, 4.2–9.3), respectively. The objective response rate was 5.0% (3/60; exact 95% CI, 1.0–13.9%) and the disease control rate was 21.7% (13/60; exact 95% CI, 12.1–34.2%); ten patients (16.7%) were not radiologically evaluable. In 21 patients aged >65 years treated in the first- or second-line setting, median PFS and OS were 3.2 and 7.7 months. Treatment-related adverse events were documented in 16 patients (26.7%), with no grade ≥3 events recorded. **Conclusions:** Intermittent low-dose MCP showed limited antitumor activity but favorable clinician-documented short-term tolerability in this multicenter cohort. Alongside prospective evidence, the results do not support routine use in unselected patients. MCP may retain an individualized palliative role when established systemic therapy is unsuitable.

## Simple Summary

For patients with advanced soft-tissue sarcoma who cannot receive standard chemotherapy, balancing potential benefit and tolerability is challenging. Oral metronomic cyclophosphamide is sometimes considered, but its value in routine care remains uncertain. We reviewed the outcomes of 60 patients treated with an intermittent low-dose schedule at three French centers. Tumor shrinkage was uncommon, and disease control was generally brief, including in older patients treated in the first- or second-line setting. Clinician-documented treatment-related adverse events were mild or moderate, with no severe events recorded. Differences in dose and accompanying medication provide context for earlier, more encouraging reports. These findings help define a selective palliative role for this convenient oral regimen when established treatments are unsuitable, rather than supporting its routine use in unselected patients.

## 1. Introduction

Soft-tissue sarcomas (STSs) are rare and heterogeneous malignancies, with an estimated annual incidence of approximately 4–5 cases per 100,000 inhabitants in Europe [1]. Despite advances in histological classification and molecular characterization, doxorubicin-based chemotherapy remains the reference first-line systemic treatment for most patients with advanced STS [1,2,3]. Its clinical benefit is modest and treatment-related toxicity, including anthracycline-associated cardiotoxicity, may limit its use in selected patients [4]. Nearly half of patients with STS are diagnosed after the age of 65 years [5]. In older patients, comorbidities, organ dysfunction, poor performance status, and geriatric vulnerabilities can further restrict access to conventional chemotherapy and complicate treatment selection [6,7,8].

Metronomic chemotherapy involves repeated administration of cytotoxic agents at relatively low doses with short or absent drug-free intervals. Initially developed as an antiangiogenic strategy, it is now regarded as a multitarget approach that may affect tumor vasculature, immune regulation, and tumor cells [9,10,11,12]. Cyclophosphamide is among the most extensively investigated drugs in metronomic regimens. Clinical studies, case reports, and reviews have explored its use across several solid tumors, including breast cancer, prostate cancer, and melanoma [13,14,15,16,17,18,19]. Mechanistic and translational studies have also described antiangiogenic, immunomodulatory, and resistance-related effects of low-dose cyclophosphamide [20,21,22,23,24].

In STS, however, evidence supporting metronomic cyclophosphamide (MCP) remains limited. The most influential report was published by Mir et al. in 2011 and included 26 patients aged 65 years or older with unresectable or metastatic STS treated with oral cyclophosphamide plus prednisolone as first- or second-line therapy. The objective response rate was 26.9%, the disease control rate was 69.2%, and median progression-free survival (PFS) was 6.8 months. In addition, radiation-associated sarcomas (RASs) appeared to derive particular benefit, accounting for five of the seven objective responses [25].

An earlier conference report of the same Mir cohort described six radiation-associated sarcomas, including five partial responses [26].

Prospective evidence in sarcoma remains limited. In a single-arm phase II study, Quek et al. treated 23 patients with advanced sarcoma using oral cyclophosphamide 50 mg once daily; no objective response was observed, the 12-week clinical benefit rate was 17%, and median PFS was 1.3 months [27]. More recently, the randomized METROPHOLYS trial compared MCP with doxorubicin as first-line treatment in older patients with advanced STS [28]. Results reported in a 2025 conference abstract suggested limited MCP activity; the trial was stopped prematurely because of recruitment difficulties. Together, these reports leave substantial uncertainty regarding MCP activity in routine practice.

We therefore conducted a multicenter retrospective study to evaluate the activity and documented safety of a previously established intermittent MCP regimen in advanced STS, including a descriptive comparison with the older first-/second-line cohort reported by Mir et al. [25].

## 2. Materials and Methods

### 2.1. Study Design and Population

We performed a retrospective multicenter electronic medical record review (ClinicalTrials.gov identifier: NCT06216990) among adults aged ≥18 years with unresectable or metastatic STS. The predefined case search covered 1 January 2005 through 31 December 2021 at three French centers (Centre Hospitalier Universitaire Jean Minjoz, Besançon; Centre Hospitalier du Mittan, Montbéliard; and Centre Georges-François Leclerc, Dijon). Patients with bone sarcomas, chondrosarcomas, desmoid tumors, or gastrointestinal stromal tumors were outside the eligibility criteria. Radiation-associated sarcomas were classified according to the Cahan criteria and subsequent modifications [29,30]. The study is reported in accordance with the STROBE statement [31] (File S1).

### 2.2. Treatment

The treatment regimen consisted of oral cyclophosphamide 50 mg twice daily on days 1–7 of repeated 14-day cycles (seven days on treatment followed by seven days off). Treatment exposure, prescribed duration, dose adjustments, completed cycles, and reasons for discontinuation were recorded.

### 2.3. Outcomes and Assessments

Tumor response was assessed from available imaging in accordance with RECIST version 1.1 [32], using local reports. The primary endpoint was PFS, measured from MCP initiation to documented radiological progression or death from any cause. Patients without progression or death were censored at the last evaluable radiological assessment. OS was measured from treatment initiation to death from any cause, with living patients censored at the last documented follow-up. Secondary endpoints were ORR (complete or partial response), DCR (complete or partial response or radiological stable disease lasting at least 12 weeks), safety, and exploratory subgroup outcomes. Patients without adequate radiological assessment were classified as non-evaluable for response and included in the denominators of ORR and DCR, without being counted as responders. Adverse events were retrospectively extracted from treating physicians’ consultation reports and medical records, and investigators assigned CTCAE version 5.0 grades from documented symptoms and available numerical laboratory values.

For a descriptive cross-cohort comparison with Mir et al. [25], we selected patients aged >65 years who had received MCP as first- or second-line systemic treatment.

### 2.4. Data Collection

Baseline data included age, ECOG performance status, dates of initial and advanced disease diagnoses, histological subtype and grade, tumor location, radiotherapy history, and status of radiation-associated sarcoma. Treatment variables included prescribed MCP dose and schedule, concomitant prednisolone, estimated treatment duration, reasons for discontinuation, cycle count, treatment line, and prior anthracycline exposure. Outcomes included best radiological response, dates of progression, death, and last follow-up, and clinician-documented adverse events. Data were extracted from medical records in the participating centers. The same inclusion criteria and endpoint definitions were applied, although the patient-identification strategies differed by center. At Besançon and Montbéliard, a hospital pharmacist systematically identified prescriptions for the relevant cyclophosphamide protocol using the electronic prescribing system shared by both sites. At Dijon, potentially eligible cases were identified by the medical oncology team based on its knowledge of treated patients, followed by on-site medical record review. The database was locked on 4 August 2022.

### 2.5. Statistical Analysis

Continuous variables were summarized with medians, interquartile ranges, and observed ranges; categorical variables were reported as counts and percentages. ORR and DCR were calculated with the full enrolled population as denominator, and exact two-sided 95% binomial confidence intervals (CIs) were calculated by the Clopper–Pearson method. Progression-free and overall survival were estimated using the Kaplan–Meier method. Median survival times and survival probabilities at three and six months were reported with 95% confidence intervals. The association between outcomes and baseline characteristics was assessed with Cox proportional hazard models, and HR with 95% CIs were provided; variables with *p* < 0.10 on univariable analysis were considered for multivariable modeling. A swimmer plot was provided to get an overview of treatment duration, follow-up and main characteristics of patients according to the treatment group. Follow-up duration was summarized descriptively as the observed time from MCP initiation to death or last documented follow-up. All tests were two-sided. *p*-values below 0.05 were considered statistically significant in these exploratory analyses, without adjustment for multiple testing. Statistical analyses were performed using SAS version 9.4 (SAS Institute Inc., Cary, NC, USA) and R software version 4.3.1 (R Development Core Team, Vienna, Austria; http://www.r-project.org).

### 2.6. Use of Generative Artificial Intelligence

Generative artificial intelligence (GenAI) was used to assist with manuscript structuring and language refinement, and the preparation of submission materials. ChatGPT (OpenAI, GPT-5.6 Sol) was used for these purposes. No GenAI tool was used to generate, analyze, or modify the study data or statistical results. All AI-assisted output was reviewed and edited by the authors.

## 3. Results

### 3.1. Patient Characteristics

**Figure 1:**
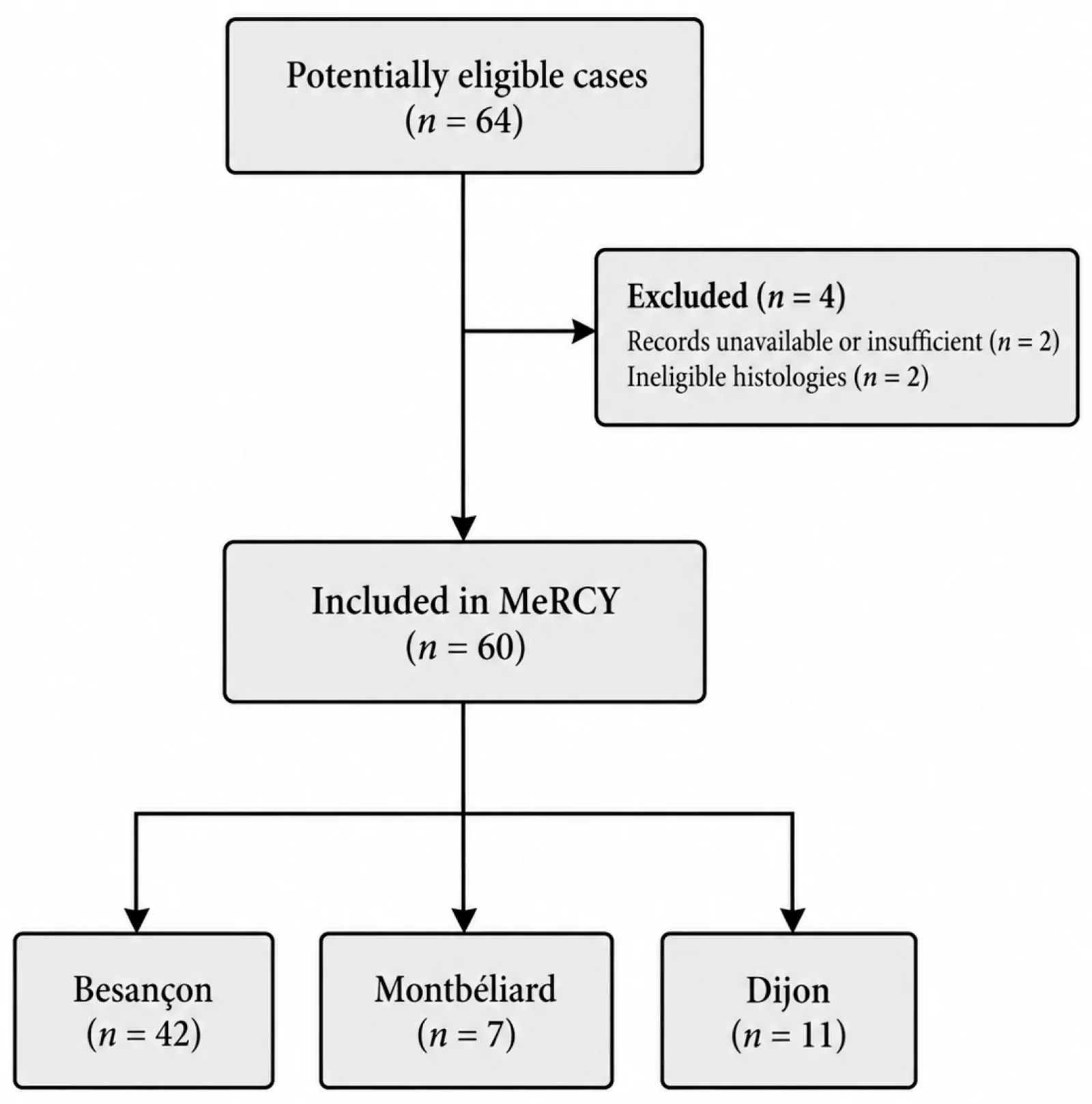
Patient identification and selection flow across the three participating centers.

Of 64 potentially eligible cases, four were excluded: two had insufficient accessible records for inclusion or data extraction (one case followed primarily in Strasbourg and one Dijon case not retrievable from the local database), and two had histologies outside the predefined study scope (one Ewing sarcoma and one gastrointestinal stromal tumor). The final cohort comprised 60 patients treated at Besançon (*n* = 42), Montbéliard (*n* = 7), and Dijon (*n* = 11). The first included patient-initiated MCP on 31 January 2008; no earlier treated patient was identified in the predefined search period (Figure 1).

Median age at treatment initiation was 70 years (range, 20–89), and 32 patients (53.3%) were female. ECOG performance status was 2–3 in 32 of 58 patients with available data (55.2%). Undifferentiated pleomorphic sarcoma, leiomyosarcoma, and liposarcoma were the most frequent histologies, together accounting for 65% of cases. Ten patients (16.7%) had radiation-associated sarcomas. Thirty-three patients started MCP in 2008–2014 and 27 in 2015–2021; ECOG 2–3 was recorded in 17/31 (54.8%) and 15/27 (55.6%) patients with available data, respectively.

Twenty-eight patients (46.7%) received MCP in the first- or second-line setting, including 21 aged >65 years. The median MCP treatment line was three. Prior anthracycline exposure was documented in 51 of 58 patients (87.9%) with known status. Median recorded treatment duration was 2.7 months. Concomitant prednisolone 20 mg once daily during the seven cyclophosphamide treatment days was administered with antitumor intent to 12 patients (20.0%): all 11 patients treated at Dijon and one Besançon patient whose treatment had begun in Dijon. The remaining 48 patients received MCP without antitumor prednisolone. According to the investigators, indications for MCP—usually after conventional options had been exhausted or when standard chemotherapy was considered unsuitable—remained broadly similar throughout the observation period.

Treatment discontinuation was primarily related to disease progression (77%). Following MCP discontinuation, 30% of patients received additional systemic treatment (Table S1). Individual treatment duration and follow-up are summarized in Figure S2.

Baseline characteristics, prior treatment, and MCP administration are summarized in Table 1 and Table 2. A descriptive comparison of the older first-/second-line MeRCY subgroup with the cohort reported by Mir et al. [25,26] is provided in Table 3.

### 3.2. Efficacy

**Figure 2:**
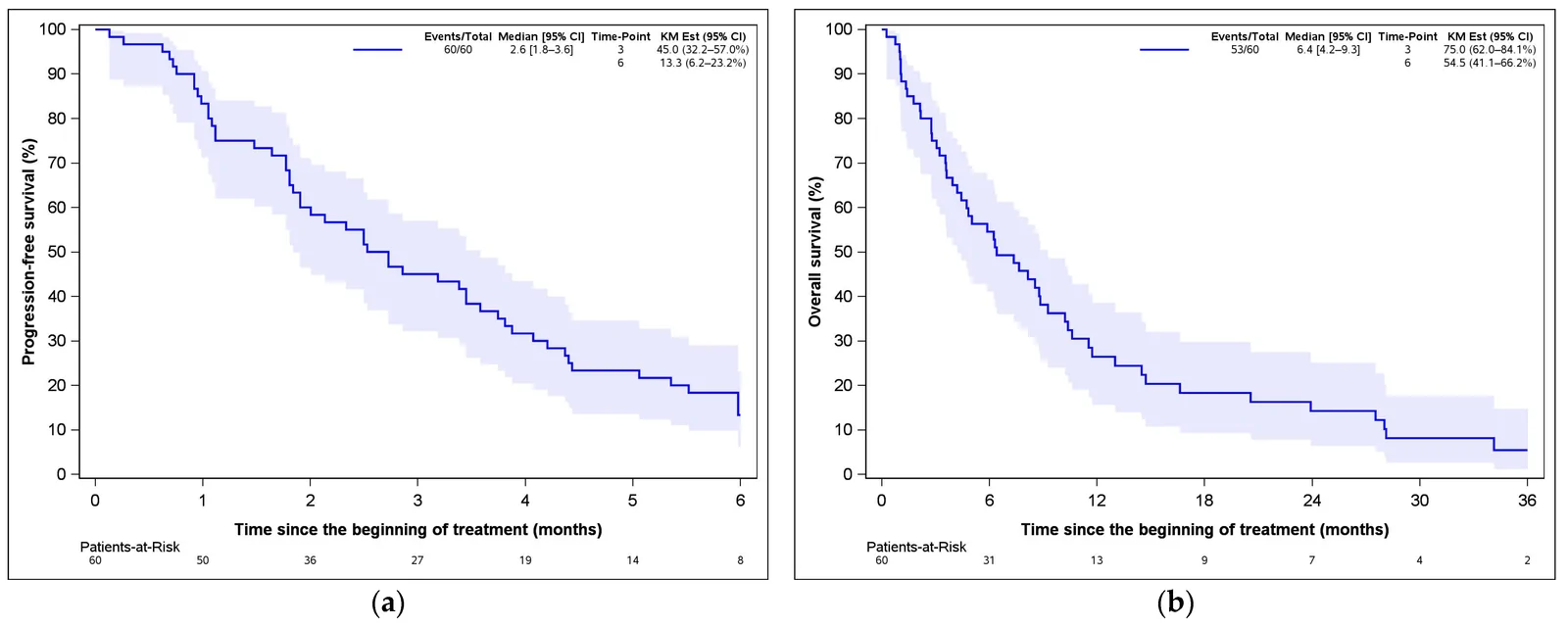
Kaplan–Meier estimates of (**a**) progression-free survival and (**b**) overall survival in the overall cohort (*n* = 60).

In the overall population, median PFS was 2.6 months (95% CI, 1.8–3.6), while median OS was 6.4 months (95% CI, 4.2–9.3) (Figure 2).

At database lock (4 August 2022), 53 patients had died and seven were censored for OS; median observed time from MCP initiation to death or last documented follow-up was 6.3 months (IQR, 2.9–11.4 months).

The best recorded radiological response was progressive disease in 37 patients (61.7%), stable disease lasting ≥12 weeks in 10 (16.7%), and partial response in three (5.0%); no complete response was observed. Ten patients (16.7%) were radiologically non-evaluable owing to the absence of a suitable RECIST assessment, mostly associated with early death or treatment discontinuation. ORR was 5.0% (3/60; exact 95% CI, 1.0–13.9%) and DCR was 21.7% (13/60; exact 95% CI, 12.1–34.2%).

Progression-free and overall survival estimates for the overall subgroup of patients receiving MCP as first- or second-line therapy are shown in Figure S1.

**Figure 3:**
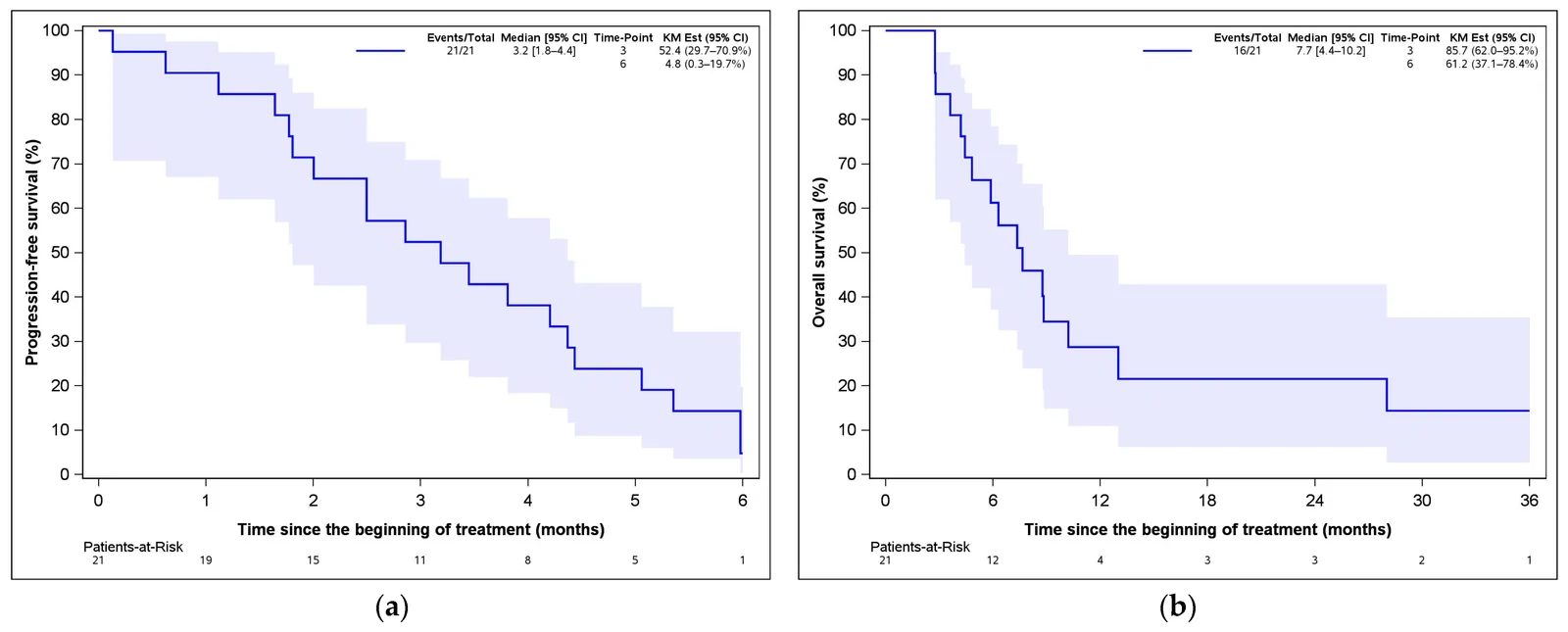
Kaplan–Meier estimates of (**a**) progression-free survival and (**b**) overall survival in patients aged >65 years treated with MCP in the first- or second-line setting (*n* = 21).

Patients aged >65 years treated with MCP as first- or second-line therapy (*n* = 21) had median PFS of 3.2 months (95% CI, 1.8–4.4) and median OS of 7.7 months (95% CI, 4.4–10.2). ORR was 9.5% (2/21; exact 95% CI, 1.2–30.4%) and DCR was 28.6% (6/21; exact 95% CI, 11.3–52.2%) (Figure 3).

**Figure 4:**
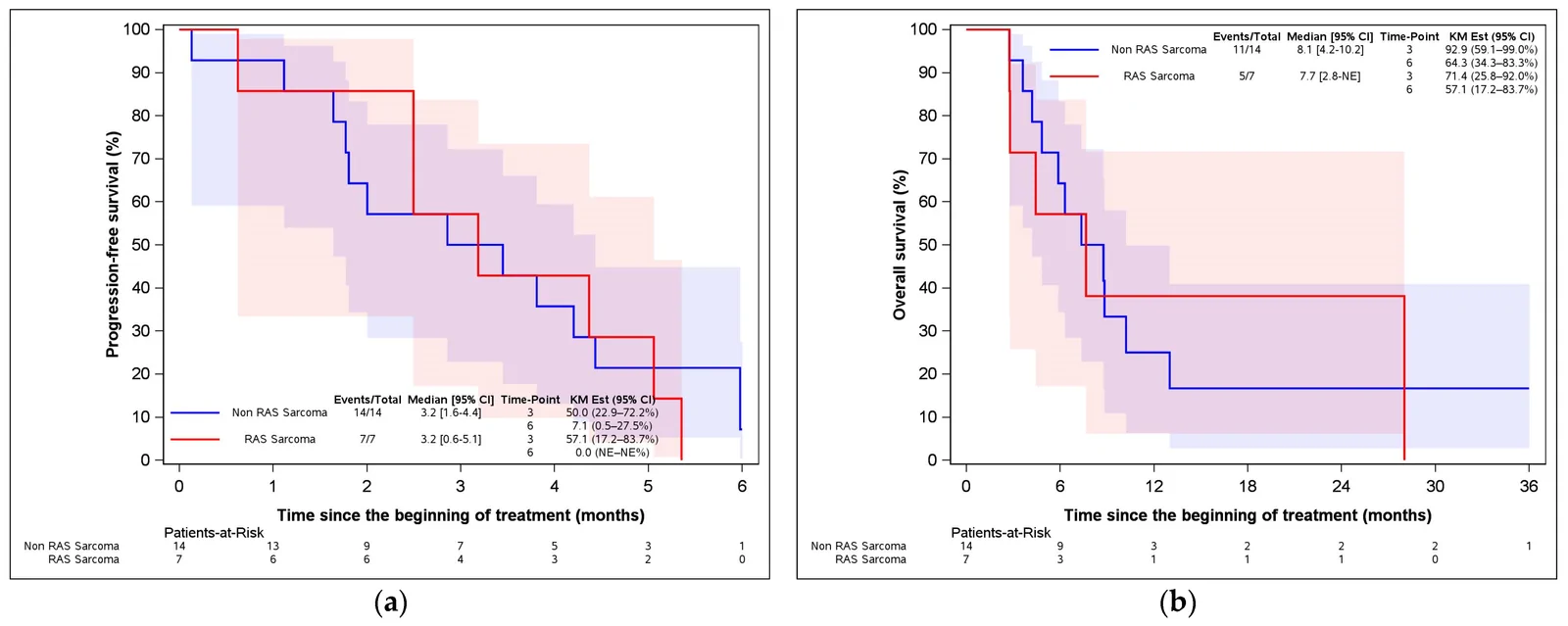
Kaplan–Meier estimates according to prior irradiation of the sarcoma field in patients aged >65 years receiving first- or second-line MCP (*n* = 21): radiation-associated sarcomas (*n* = 7) versus other STS (*n* = 14). (**a**) Progression-free survival; (**b**) overall survival. RAS denotes radiation-associated sarcoma; Non-RAS denotes other STS.

Within the older first-/second-line subgroup, seven patients had radiation-associated sarcomas. Figure 4 compares these seven patients with the 14 patients with other sarcomas. No statistically significant difference in PFS or OS was observed in this exploratory comparison.

### 3.3. Safety

Treatment-related adverse events were documented in 16 patients (26.7%). All recorded events were retrospectively assigned grades 1–2; no grade ≥3 treatment-related adverse events were identified in the clinical records reviewed.

The most frequently documented events were nausea/vomiting (10%), asthenia (8%), and anemia (7%). No febrile neutropenia, thrombocytopenia, or treatment-related death was reported in the extracted clinical records. Neutropenia and lymphopenia were not explicitly recorded as treatment-related adverse events.

Only two patients (3%) required dose modification because of toxicity. Both cases were related to clinical rather than biological adverse events and improved following treatment adjustment (Table S2).

### 3.4. Exploratory Prognostic Analyses

No baseline characteristic was significantly associated with PFS in either the overall population or predefined subgroups.

For OS, histological grade was the only variable independently associated with outcome: compared with grade I tumors, grade III sarcomas were associated with significantly shorter survival in multivariable analysis (HR 2.92, 95% CI 1.15–7.56; *p* = 0.024).

Neither previously irradiated-field status nor corticosteroid co-administration was associated with survival outcomes.

Detailed exploratory analyses are reported in Tables S3–S8.

## 4. Discussion

### 4.1. Main Findings

To our knowledge, MeRCY is the largest independent multicenter real-world evaluation of intermittent low-dose MCP in advanced STS. In the full cohort, ORR was 5.0% and median PFS was 2.6 months; in the older first-/second-line subgroup, the corresponding values were 9.5% and 3.2 months. These findings indicate limited radiological antitumor activity, while clinician-documented short-term tolerability was favorable.

Overall, the activity observed was below that of the initial encouraging retrospective series, without suggesting that the regimens or populations were directly interchangeable.

### 4.2. Comparison with Previous Studies of Metronomic Cyclophosphamide

Mir et al. reported an ORR of 26.9%, DCR of 69.2%, and median PFS of 6.8 months in 26 older patients treated in the first- or second-line setting [25]. Corresponding estimates in the MeRCY comparison subgroup (***n*** = 21) were 9.5%, 28.6%, and 3.2 months. Table 3 presents patient characteristics and treatment protocols side by side. Patients in MeRCY were older (median age, 80 versus 72 years) and more frequently exposed to anthracyclines (13/20 [65.0%] versus 6/26 [23.1%]); 17/20 (85.0%) patients with available performance-status data had ECOG 2–3. Differences in pretreatment, baseline fitness, patient selection, and cyclophosphamide dose may limit direct comparisons with findings from earlier studies.

Other prospective data are more consistent with the modest activity observed in our cohort. In the single-arm phase II study by Quek et al., 23 treated patients with advanced sarcoma had no objective response, a 12-week clinical benefit rate of 17%, and a median PFS of 1.3 months [27]. In METROPHOLYS, the ORR with MCP was 6.7% and median PFS was 2.3 months [28]. Although cross-study comparisons are inherently limited by differences in eligibility criteria, line of treatment, histological composition, and endpoint definitions, these independent prospective observations support a more conservative estimate of MCP activity than that suggested by the initial series.

Treatment-regimen differences also warrant consideration. Both studies administered cyclophosphamide on days 1–7 of a 14-day cycle, but Mir et al. used 100 mg twice daily (50 mg twice daily in two patients with ECOG 3) plus prednisolone 20 mg daily during treatment weeks [25]. MeRCY used cyclophosphamide 50 mg twice daily, based on the earlier intermittent regimen reported by Ghiringhelli et al. [20] and adopted locally around 2008; the subsequent publication by Mir et al. did not result in a change in the local dose. Antitumor prednisolone use was less frequent in MeRCY and strongly associated with center, complicating interpretation of the descriptive comparison. These regimen and population differences do not establish a causal dose–response relationship. A summary of the main studies evaluating metronomic cyclophosphamide in advanced soft-tissue sarcoma is provided in Table S9.

### 4.3. Radiation-Associated Sarcomas

Radiation-associated sarcomas are uncommon and heterogeneous secondary malignancies. The relevant diagnostic framework derives from the Cahan criteria and subsequent modifications; contemporary studies highlight varied clinicopathological presentations and often unfavorable outcomes [29,30,33].

Only ten patients in our full cohort, including seven in the older first-/second-line subgroup, had radiation-associated sarcomas. Although no association with improved outcomes was detected, this small subgroup cannot exclude activity in a specific biological context. Our findings therefore do not support radiation-associated sarcoma status alone as a clinically useful predictor of MCP benefit.

### 4.4. Integration of Prospective Evidence and Feasibility of Randomized Trials

The randomized METROPHOLYS trial provides prospective context [28]. According to the published 2025 conference abstract, 42 patients were randomized from a planned 132; 21 patients in the MCP arm and 18 in the doxorubicin arm received treatment. The reported ORR was 6.7% versus 30.8%, median PFS was 2.3 versus 4.1 months, and median OS was 20.9 versus 19.1 months, respectively. Recruitment was discontinued because of insufficient accrual, limiting statistical power and preventing definitive comparative conclusions.

Recruitment difficulties have also affected other prospective geriatric sarcoma studies. The French UNICANCER GERICO14 randomized trial, designed to compare oral cyclophosphamide with doxorubicin in older patients with advanced STS excluding rhabdomyosarcomas, was discontinued because of insufficient accrual [34]. These experiences illustrate the challenges of conducting prospective studies in this heterogeneous and often frail population.

### 4.5. Clinical Implications

Older or frail patients with advanced STS may be unable to receive standard anthracycline-based treatment because of comorbidity, organ dysfunction, or performance status [6,8]. Our findings do not support MCP as a substitute for standard first-line therapy in patients fit to receive it, or its routine use in unselected patients with advanced STS. Nevertheless, intermittent oral MCP may remain a pragmatic, individualized palliative option when established systemic therapy is unsuitable and treatment intensity is a major consideration. As patient-reported outcomes and formal quality-of-life measures were not collected, any quality-of-life advantage remains unproven.

### 4.6. Safety Considerations

Treatment-related adverse events were documented in 16/60 patients (26.7%), all graded 1–2 in the available clinical records, and only two patients required a dose modification. No grade ≥3 treatment-related toxicity was recorded, supporting a favorable documented short-term tolerability profile. Although clinically significant events would be expected to prompt medical documentation, the absence of an independent systematic review of laboratory results may have reduced ascertainment of asymptomatic hematological abnormalities.

No secondary hematological malignancy was observed in this cohort. Rare late complications of prolonged alkylating-agent exposure cannot, however, be excluded by a study characterized by short treatment duration and limited survival. A therapy-related acute leukemia has been reported after cyclophosphamide-based metronomic chemotherapy in a heavily pretreated patient, although causal attribution in that setting is uncertain [35]. These observations support caution when extrapolating short-term tolerability to long-term safety.

### 4.7. Strengths and Limitations

The principal strengths of this study are its multicenter design, its inclusion of a real-world population with a high prevalence of poor performance status, and its independent assessment of previously reported efficacy signals. The older first-/second-line subgroup provides a clinically interpretable, albeit descriptive, comparison with the cohort reported by Mir et al. The use of consistent eligibility criteria and endpoint definitions across the three centers is an additional strength.

Several limitations should be acknowledged. The retrospective, non-comparative design is subject to selection bias, confounding by indication, and incomplete ascertainment of some adverse events. Case identification relied on pharmacy prescribing records at Besançon and Montbéliard but on clinical case identification at Dijon, which could affect completeness. Although prescribing indications remained broadly stable, the long observation period (2008–2021) may have introduced changes in supportive care, imaging practice, pathological classification, and treatment sequencing. Histological heterogeneity reflects routine STS practice but may obscure subtype-specific activity; histological grade was unavailable in 13 patients. Small subgroup sizes limit precision and increase the risk of unstable estimates. Differences in MCP dose, concomitant prednisolone, and previous treatment limit cross-study comparability. Laboratory toxicity was assessed from existing clinical documentation rather than by independent systematic reassessment. Finally, patient-reported outcomes and pharmacokinetic, pharmacodynamic, immune, or other translational analyses were unavailable to identify predictors of benefit or resistance.

## 5. Conclusions

In this multicenter retrospective cohort, intermittent low-dose metronomic cyclophosphamide showed limited antitumor activity in advanced STS, including among older patients receiving it in the first- or second-line setting. Together with prospective observations, these results do not favor routine MCP treatment in unselected patients or its substitution for standard chemotherapy in patients fit to receive it. Short-term tolerability appeared favorable, with few dose modifications and no grade ≥3 treatment-related adverse events documented by clinicians. MCP may retain a role as an individualized palliative option for patients unable to receive conventional systemic treatment.

## Figures and Tables

**Table 1:** Characteristics of the study population.

	Total	Line of Treatment and Age at MCP Initiation	
		L1–L2 Overall	L1–L2 > 65 Years
**Sample size, No.**	**60**	**28**	**21**
**Sex, No. (%)**			
Men	28 (47%)	14 (50%)	8 (38%)
Women	32 (53%)	14 (50%)	13 (62%)
**Age at time of unresectable diagnosis, years**			
Median (IQR)	66 (53–77)	76 (59–80)	79 (76–82)
Min–max	19–89	19–89	53–89
**ECOG PS, No. (% of available)**			
0	4 (7%)	3 (11%)	1 (5%)
1	22 (38%)	6 (22%)	2 (10%)
2	24 (41%)	13 (48%)	12 (60%)
3	8 (14%)	5 (19%)	5 (25%)
Unknown	2	1	1
**Stage at diagnosis, No. (%)**			
Unresectable or metastatic at diagnosis	27 (45%)	15 (54%)	11 (52%)
Metachronous metastases	33 (55%)	13 (46%)	10 (48%)
**Number of metastatic sites**			
0	16 (27%)	11 (39%)	9 (43%)
1	17 (28%)	9 (32%)	6 (29%)
>1	27 (45%)	8 (29%)	6 (29%)
Median (IQR)	1 (0–2.5)	1 (0–2)	1 (0–2)
Min–max	0–9	0–4	0–4
**Primary site, No. (%) ^(1)^**			
Limbs	21 (35%)	8 (29%)	2 (10%)
Retroperitoneum	11 (18%)	5 (18%)	4 (19%)
Chest Wall	6 (10%)	4 (14%)	4 (19%)
Other	22 (37%)	11 (39%)	11 (52%)
**Histological subtypes, No. (%)**			
Undifferentiated Pleomorphic Sarcoma	15 (25%)	8 (29%)	7 (33%)
Liposarcoma	12 (20%)	6 (21%)	4 (19%)
Leiomyosarcoma	12 (20%)	2 (7%)	2 (10%)
Angiosarcoma	6 (10%)	3 (11%)	3 (14%)
Synovial sarcoma	5 (8%)	4 (14%)	2 (10%)
Other	10 (17%)	5 (18%)	3 (14%)
Alveolar sarcoma	1	0	0
Alveolar rhabdomyosarcoma	1	1	0
Fusiform cell sarcoma	1	0	0
Histiocytic sarcoma	1	1	1
Solitary fibrous tumor	2	1	0
Fibrosarcoma	1	1	1
Myxofibrosarcoma	1	0	0
Neurofibrosarcoma	1	1	1
UTROSCT	1	0	0
**Grade, No. (% of available)**			
Grade I	13 (28%)	5 (22%)	4 (21%)
Grade II	15 (32%)	7 (30%)	5 (26%)
Grade III	19 (40%)	11 (48%)	10 (53%)
Unknown	13	5	2
**Radiation-associated Sarcoma, No. (%)**			
No	50 (83%)	21 (75%)	14 (67%)
Yes	10 (17%)	7 (25%)	7 (33%)
**Time from initial diagnosis until unresectable diagnosis, Months**			
Median (IQR)	5.6 (0.0–26.2)	0.6 (0.0–28.9)	1.0 (0.0–2.0)
Min–max	0–379	0–379	0.0–4.0
**Time from initial diagnosis until unresectable diagnosis, Months (metachronous only)**			
Median (IQR)	24.3 (8.0–36.0)	26.2 (6.3–36.0)	19.3 (6.3–63.6)
Min–max	0.2–158.2	0.2–71.0	1.0–71.0

^(1)^ Percentages may not total 100 due to rounding; L1–L2 = first- or second-line treatment; IQR = Interquartile Range; UTROSCT = Uterine Tumor Resembling Ovarian Sex Cord Tumor; ECOG PS = Eastern Cooperative Oncology Group—Performance Status.

**Table 2:** Treatment modalities.

	Total	Line of Treatment and Age at MCP Initiation	
		L1–L2 Overall	L1–L2 > 65 Years
**Sample size, No.**	60	28	21
**Primary tumor surgery, No. (%)**			
Yes	34 (57%)	14 (50%)	10 (48%)
No	26 (43%)	14 (50%)	11 (52%)
**Pathological residual status, No. (%) ^(1)^**			
R0	18 (62%)	6 (50%)	4 (44%)
R1/R2	11 (37%)	6 (50%)	5 (56%)
Unknown	31	16	12
**Prior radiotherapy, No. (%)**			
Yes	34 (57%)	14 (50%)	9 (43%)
No	26 (43%)	14 (50%)	12 (57%)
**Radiotherapy indications ^(2)^, No. (%) ^(1)^**			
Primary Tumor (Neo-adjuvant)	1 (3%)	1 (8%)	1 (9%)
Primary Tumor (Adjuvant)	12 (38%)	4 (31%)	3 (27%)
Primary Tumor (Local recurrence)	1 (3%)	0 (0%)	0 (0%)
Metastasis (Pain-control)	8 (25%)	1 (8%)	0 (0%)
Other Primary Tumor	10 (31%)	7 (54%)	7 (64%)
Unknown	2	1	1
**MCP treatment line ^(3)^, No. (%) ^(1)^**			
1	6 (10%)	6 (21%)	6 (29%)
2	22 (37%)	22 (79%)	15 (71%)
3	15 (25%)	-	-
4	13 (22%)	-	-
5	1 (2%)	-	-
6	2 (3%)	-	-
8	1 (2%)	-	-
Median (IQR)	3 (2–4)	2 (2–2)	2 (1–2)
Min–max	1–8	1–2	1–2
**Anthracycline administration, No. (% of available)**			
Yes	51 (88%)	20 (74%)	13 (65%)
No	7 (12%)	7 (26%)	7 (35%)
Unknown	2	1	1
**Age at MCP initiation, Years (range)**			
Median (IQR)	70 (58–79)	78 (68–83)	80 (77–84)
Min–max	20–89	20–89	72–89
**Time from unresectable diagnosis until MCP initiation, Months**			
Median (IQR)	15.0 (6.0–27.7)	6.9 (4.5–17.8)	7.5 (4.4–18.9)
Min–max	0.0–69.9	0.0–60.0	0.0–60.0
**Number of full MCP cycles, No. (%) ^(1)^**			
<1	4 (7%)	2 (7%)	1 (5%)
1	16 (27%)	9 (32%)	6 (29%)
2	12 (20%)	2 (7%)	2 (10%)
3	11 (18%)	8 (29%)	6 (29%)
4	7 (12%)	1 (4%)	1 (5%)
5	3 (5%)	1 (4%)	1 (5%)
6	4 (7%)	4 (14%)	4 (19%)
7	1 (2%)	1 (4%)	0 (0%)
8	1 (2%)	0 (0%)	0 (0%)
28	1 (2%)	0 (0%)	0 (0%)
Median (IQR)	2 (1–4)	3 (1–4)	3 (1–4)
Min–max	0–28	0–7	0–6
**Total MCP dose, Milligrams (range)**			
Median (IQR)	3933 (1979–6538)	4184 (2205–6538)	4359 (3107–6664)
Min–max	200–42,888	200–10,171	200–10,171
**MCP treatment duration, Months (range)**			
Median (IQR)	2.7 (1.3–4.4)	3.0 (1.4–4.4)	3.2 (2.3–4.4)
Min–max	0.1–28.1	0.1–7.0	0.1–6.7
**Concomitant corticosteroid use, No. (%)**			
Yes	12 (20%)	6 (21%)	4 (19%)
No	48 (80%)	22 (79%)	17 (81%)

^(1)^ Percentages may not total 100 due to rounding. ^(2)^ Some patients received radiotherapy for multiple indications. ^(3)^ Perioperative chemotherapy included; L1–L2 = first- or second-line treatment; IQR = Interquartile Range; MCP = Metronomic Cyclophosphamide.

**Table 3:** Descriptive comparison of patients from Mir et al. (2011) [25] and the MeRCY older first-/second-line subgroup.

Characteristic	Mir et al. [25,26] (*n* = 26)	MeRCY Subgroup (*n* = 21)
**Patients treated (*n*)**	26	21
**Setting**	Single-center retrospective	Three-center retrospective subgroup
**Median age at MCP initiation, years (range)**	72 (66–88)	80 (72–89)
**Women, *n* (%)**	20 (76.9%)	13 (61.9%)
**ECOG Performance Status, median (range)**	2 (1–3)	2 (0–3); 1 missing
**First-line/second-line MCP, *n***	19/7	6/15
**Prior anthracycline exposure, *n* (%)**	6/26 (23.1%)	13/20 (65.0%); 1 missing
**Radiation associated sarcoma (RAS), *n* (%)**	6 (23.1%)	7 (33.3%)
**Grade III, *n* (%)**	17/26 (65.4%)	10/19 (52.6%); 2 missing
**Undifferentiated or poorly differentiated sarcoma, *n* (%)**	8 (30.8%)	7 UPS (33.3%)
**Liposarcoma, *n* (%)**	5 (19.2%)	4 (19.0%)
**Leiomyosarcoma, *n* (%)**	5 (19.2%)	2 (9.5%)
**Angiosarcoma, *n* (%)**	3 (11.5%)	3 (14.3%)
**Median number of MCP cycles (range)**	10 (2–41)	3 (0–6)
**Cyclophosphamide regimen**	100 mg twice daily, days 1–7/14	50 mg twice daily, days 1–7/14
**Prednisolone**	20 mg/day, days 1–7/14 days; all patients	20 mg/day, days 1–7/14 days; 4/21 (19.0%)
**ORR**	7/26 (26.9%)	2/21 (9.5%)
**DCR (≥12 weeks)**	18/26 (69.2%)	6/21 (28.6%)
**Median PFS, months**	6.8	3.2
**Median OS, months**	14.1 [26]	7.7

MeRCY efficacy percentages use all 21 patients in the subgroup; for baseline characteristics, available-case denominators and missing observations are shown explicitly. The Mir cohort results derive from the 2011 publication and its 2010 conference report [25,26].

## Data Availability

The data presented in this study are available on reasonable request from the corresponding author. Individual-level data are not publicly available due to privacy and data-protection restrictions.

## Supplementary Materials

The following supporting information can be downloaded at: https://www.mdpi.com/article/10.3390/cancers18213497/s1, Figure S1: Progression-free survival and overall survival in patients treated with metronomic cyclophosphamide as first- or second-line therapy; Figure S2: Swimmer plot; Table S1: Reasons for metronomic cyclophosphamide discontinuation and subsequent systemic treatment; Table S2: Treatment-related adverse events during metronomic cyclophosphamide therapy; Table S3: Univariable Cox regression analysis of progression-free survival in the overall population; Table S4: Univariable and multivariable Cox regression analyses of overall survival in the overall population; Table S5: Univariable Cox regression analysis of progression-free survival in patients treated with metronomic cyclophosphamide as first- or second-line therapy; Table S6: Univariable Cox regression analysis of overall survival in patients treated with metronomic cyclophosphamide as first- or second-line therapy; Table S7: Univariable Cox regression analysis of progression-free survival in patients aged >65 years treated with metronomic cyclophosphamide as first- or second-line therapy; Table S8: Univariable Cox regression analysis of overall survival in patients aged >65 years treated with metronomic cyclophosphamide as first- or second-line therapy; Table S9: Comparison of studies evaluating metronomic cyclophosphamide in advanced soft-tissue sarcoma. File S1. STROBE Reporting Checklist—Cohort Study.

## Abbreviations

The following abbreviations are used in this manuscript:

CI	Confidence interval
CTCAE	Common Terminology Criteria for Adverse Events
DCR	Disease control rate
ECOG	Eastern Cooperative Oncology Group
HR	Hazard ratio
IQR	Interquartile range
L1–L2	First- and second-line treatment
MCP	Metronomic cyclophosphamide
ORR	Objective response rate
OS	Overall survival
PFS	Progression-free survival
RECIST	Response Evaluation Criteria in Solid Tumors
STROBE	Strengthening the Reporting of Observational Studies in Epidemiology
STS	Soft-tissue sarcoma
UTROSCT	Uterine tumor resembling ovarian sex cord tumor

## References

[1] Gronchi A., Miah A.B., Dei Tos A.P., Abecassis N., Bajpai J., Bauer S., Biagini R., Bielack S., Blay J.Y., Bolle S. (2021). Soft Tissue and Visceral Sarcomas: ESMO–EURACAN–GENTURIS Clinical Practice Guidelines for Diagnosis, Treatment and Follow-Up. Ann. Oncol..

[2] Judson I., Verweij J., Gelderblom H., Hartmann J.T., Schöffski P., Blay J.-Y., Kerst J.M., Sufliarsky J., Whelan J., Hohenberger P. (2014). Doxorubicin Alone versus Intensified Doxorubicin plus Ifosfamide for First-Line Treatment of Advanced or Metastatic Soft-Tissue Sarcoma: A Randomised Controlled Phase 3 Trial. Lancet Oncol..

[3] Seddon B., Strauss S.J., Whelan J., Leahy M., Woll P.J., Cowie F., Rothermundt C., Wood Z., Benson C., Ali N. (2017). Gemcitabine and Docetaxel versus Doxorubicin as First-Line Treatment in Previously Untreated Advanced Unresectable or Metastatic Soft-Tissue Sarcomas (GeDDiS): A Randomised Controlled Phase 3 Trial. Lancet Oncol..

[4] Ferreira A.L.A., Matsubara L.S., Matsubara B.B. (2008). Anthracycline-Induced Cardiotoxicity. Cardiovasc. Hematol. Agents Med. Chem..

[5] Nijhuis P.H., Schaapveld M., Otter R., Molenaar W.M., van der Graaf W.T., Hoekstra H.J. (1999). Epidemiological Aspects of Soft Tissue Sarcomas (STS)--Consequences for the Design of Clinical STS Trials. Eur. J. Cancer.

[6] Yousaf N., Harris S., Martin-Liberal J., Stanway S., Linch M., Ifijen M., Al Muderis O., Khabra K., Fisher C., Noujaim J. (2015). First Line Palliative Chemotherapy in Elderly Patients with Advanced Soft Tissue Sarcoma. Clin. Sarcoma Res..

[7] Younger E., Litière S., Le Cesne A., Mir O., Gelderblom H., Italiano A., Marreaud S., Jones R.L., Gronchi A., van der Graaf W.T.A. (2018). Outcomes of Elderly Patients with Advanced Soft Tissue Sarcoma Treated with First-Line Chemotherapy: A Pooled Analysis of 12 EORTC Soft Tissue and Bone Sarcoma Group Trials. Oncologist.

[8] Dale W., Klepin H.D., Williams G.R., Alibhai S.M.H., Bergerot C., Brintzenhofeszoc K., Hopkins J.O., Jhawer M.P., Katheria V., Loh K.P. (2023). Practical Assessment and Management of Vulnerabilities in Older Patients Receiving Systemic Cancer Therapy: ASCO Guideline Update. J. Clin. Oncol..

[9] Kerbel R.S., Kamen B.A. (2004). The Anti-Angiogenic Basis of Metronomic Chemotherapy. Nat. Rev. Cancer.

[10] Pasquier E., Kavallaris M., André N. (2010). Metronomic Chemotherapy: New Rationale for New Directions. Nat. Rev. Clin. Oncol..

[11] André N., Carré M., Pasquier E. (2014). Metronomics: Towards Personalized Chemotherapy?. Nat. Rev. Clin. Oncol..

[12] Cazzaniga M.E., Cordani N., Capici S., Cogliati V., Riva F., Cerrito M.G. (2021). Metronomic Chemotherapy. Cancers.

[13] Colleoni M., Rocca A., Sandri M.T., Zorzino L., Masci G., Nolè F., Peruzzotti G., Robertson C., Orlando L., Cinieri S. (2002). Low-Dose Oral Methotrexate and Cyclophosphamide in Metastatic Breast Cancer: Antitumor Activity and Correlation with Vascular Endothelial Growth Factor Levels. Ann. Oncol..

[14] Glode L.M., Barqawi A., Crighton F., Crawford E.D., Kerbel R. (2003). Metronomic Therapy with Cyclophosphamide and Dexamethasone for Prostate Carcinoma. Cancer.

[15] De Pas T., Colleoni M., Orlando L., Masci G., Rocca A., Catania C., Curigliano G., Manzoni S., Goldhirsch A., de Braud F. (2005). Reply to the Article “Metronomic Therapy with Cyclophosphamide Induces Rat Lymphoma and Sarcoma Regression, and Is Devoid of Toxicity” by V. R. Rozados et al. (Ann Oncol 2004; 15: 1543–1550): … and in Humans?. Ann. Oncol..

[16] Borne E., Desmedt E., Duhamel A., Mirabel X., Dziwniel V., Maire C., Florin V., Martinot V., Penel N., Vercambre-Darras S. (2010). Oral Metronomic Cyclophosphamide in Elderly with Metastatic Melanoma. Investig. New Drugs.

[17] Penel N., Dansin E., Desauw C., Mortier L., Clisant S., Damie S., Fournier C., Degardin M., Adenis A. (2008). Oral Metronomic Cyclophosphamide (OMC) versus Megestrol Acetate (MA) among Patients with Advanced, Refractory and Progressive Solid Tumours: A Randomized Phase II Study. J. Clin. Oncol..

[18] Lien K., Georgsdottir S., Sivanathan L., Chan K., Emmenegger U. (2013). Low-Dose Metronomic Chemotherapy: A Systematic Literature Analysis. Eur. J. Cancer.

[19] Gagliato D.d.M., Linck R.D.M., Bezerra R.O.F., Souto M., Lopes G.L., Baiocchi G., Mano M.S. (2016). An Uncommon Response to Metronomic Therapy in a Heavily Pretreated Patient with Metastatic Carcinosarcoma: A Case Report. J. Med. Case Rep..

[20] Ghiringhelli F., Menard C., Puig P.E., Ladoire S., Roux S., Martin F., Solary E., Le Cesne A., Zitvogel L., Chauffert B. (2007). Metronomic Cyclophosphamide Regimen Selectively Depletes CD4^+^CD25^+^ Regulatory T Cells and Restores T and NK Effector Functions in End Stage Cancer Patients. Cancer Immunol. Immunother..

[21] Patten S.G., Adamcic U., Lacombe K., Minhas K., Skowronski K., Coomber B.L. (2010). VEGFR2 Heterogeneity and Response to Anti-Angiogenic Low Dose Metronomic Cyclophosphamide Treatment. BMC Cancer.

[22] Chow A., Francia G., Kouri A., Lee C., Ebos J.M., Kerbel R.S., Emmenegger U. (2014). Impaired Autophagy Mediates Resistance to Low-Dose Metronomic Cyclophosphamide Chemotherapy. Clin. Cancer Drugs.

[23] Abu Eid R., Razavi G.S.E., Mkrtichyan M., Janik J., Khleif S.N. (2016). Old-School Chemotherapy in Immunotherapeutic Combination in Cancer, A Low-Cost Drug Repurposed. Cancer Immunol. Res..

[24] Laheurte C., Thiery-Vuillemin A., Calcagno F., Legros A., Simonin H., Boullerot L., Jacquin M., Nguyen T., Mouillet G., Borg C. (2020). Metronomic Cyclophosphamide Induces Regulatory T Cells Depletion and PSA-Specific T Cells Reactivation in Patients with Biochemical Recurrent Prostate Cancer. Int. J. Cancer.

[25] Mir O., Domont J., Cioffi A., Bonvalot S., Boulet B., Le Pechoux C., Terrier P., Spielmann M., Le Cesne A. (2011). Feasibility of Metronomic Oral Cyclophosphamide plus Prednisolone in Elderly Patients with Inoperable or Metastatic Soft Tissue Sarcoma. Eur. J. Cancer.

[26] Mir O., Domont J., Cioffi A., Bonvalot S., Boulet B., Le Pechoux C., Missenard G., Rimareix F., Terrier P., Le Cesne A. (2010). Metronomic Oral Cyclophosphamide (CPM) and Prednisolone in Elderly Patients (Pts) with Inoperable or Metastatic Soft Tissue Sarcoma (STS). J. Clin. Oncol..

[27] Quek R., Lim S.L., Chay W.Y., Soh L.T., Tan S.H., Goh W.L., Leong M., Khoo R.C.L., Selvarajan S., Sittampalam K. (2017). Phase 2 Clinical Study of Metronomic Oral Cyclophosphamide (MOC) in Advanced Sarcomas. Ann. Oncol..

[28] Brunello A., Nannini M., Ibrahim T., Gurrieri L., Ferraresi V., Bertuzzi A.F., Vincenzi B., Grignani G., Aliberti S., Cingarlini S. (2025). 2692MO Efficacy and Safety of Metronomic Cyclophosphamide (MC) VS Doxorubicin (DOXO) as First-Line Treatment in Older Patients (PTS) with Advanced Soft Tissue Sarcomas (STS): The METROPHOLYS Trial. Ann. Oncol..

[29] Cahan W.G., Woodard H.Q. (1948). Sarcoma Arising in Irradiated Bone; Report of 11 Cases. Cancer.

[30] Murray E.M., Werner D., Greeff E.A., Taylor D.A. (1999). Postradiation Sarcomas: 20 Cases and a Literature Review. Int. J. Radiat. Oncol. Biol. Phys..

[31] von Elm E., Altman D.G., Egger M., Pocock S.J., Gøtzsche P.C., Vandenbroucke J.P., STROBE Initiative (2008). The Strengthening the Reporting of Observational Studies in Epidemiology (STROBE) Statement: Guidelines for Reporting Observational Studies. J. Clin. Epidemiol..

[32] Eisenhauer E.A., Therasse P., Bogaerts J., Schwartz L.H., Sargent D., Ford R., Dancey J., Arbuck S., Gwyther S., Mooney M. (2009). New Response Evaluation Criteria in Solid Tumours: Revised RECIST Guideline (Version 1.1). Eur. J. Cancer.

[33] Inchaustegui M.L., Kon-Liao K., Ruiz-Arellanos K., Silva G.A.E., Gonzalez M.R., Pretell-Mazzini J. (2023). Treatment and Outcomes of Radiation-Induced Soft Tissue Sarcomas of the Extremities and Trunk-A Systematic Review of the Literature. Cancers.

[34] UNICANCER Randomized Phase III Study of Oral Cyclophosphamide vs Doxorubicin in 65 Years or Older Patients with Advanced or Metastatic Soft Tissue Sarcoma: A UNICANCER/GERICO Multicenter Program. https://clinicaltrials.gov/ct2/show/NCT04757337.

[35] Dobi E., Nguyen T., Borg C., Pivot X., Royer B., Kim S. (2013). Treatment-Induced Acute Leukaemia after Major Response to Cyclophosphamide-Based Metronomic Chemotherapy in Refractory Heavily Pre-Treated Prostate Cancer. J. Cancer Ther..

