# Supplementary material for: Real-World Activity and Safety of Metronomic Cyclophosphamide in Advanced Soft-Tissue Sarcoma: The Multicenter MeRCY Study
Source: Cancers (Basel). 2026 Oct 30;18(21):3497. doi: 10.3390/cancers18213497 (PMC13761905; doi:10.3390/cancers18213497)
Supplement: Supplementary file 1 [file cancers-18-03497-s001.zip › File_S1_STROBE_Checklist.pdf]

File S1. STROBE Reporting Checklist — Cohort Study

Item	Section	STROBE reporting recommendation (paraphrased)	Location in manuscript	Manuscript-specific reporting note
1	**Title and abstract**	Identify the observational design in the title/abstract and provide an informative, balanced summary.	Main text, Abstract	The retrospective multicenter design is identified in the structured abstract, which reports the study population, treatment, efficacy, and safety findings in a balanced manner.
2	**Background / rationale**	Explain the scientific background and rationale for the investigation.	Main text, Section 1	The Introduction describes the limited evidence base, the Mir et al. efficacy signal, subsequent prospective evidence, and the need for independent external validation.
3	**Objectives**	State specific objectives, including relevant prespecified hypotheses.	Main text, Section 1	The objective is explicitly framed as an independent evaluation of MCP activity and safety and an assessment of whether previously reported efficacy signals can be reproduced in a broader real-world population.
4	**Study design**	Present key elements of the study design early in the paper.	Main text, Section 2.1	The retrospective multicenter electronic medical-record design is stated at the beginning of Materials and Methods.
5	**Setting**	Describe the setting, locations, and relevant dates, including recruitment/exposure/follow-up periods.	Main text, Sections 2.1 and 2.4	The predefined case-identification period extended from January 2005 to December 2021; included patients initiated MCP between 2008 and 2021. The study involved three French centers, and the database was locked on 4 August 2022.
6a	**Participants**	Give eligibility criteria and the sources/methods of participant selection and follow-up.	Main text, Sections 2.1, 2.4 and 3.1	Eligibility and exclusion criteria, participating centers, and medical-record sources are described. Case identification relied on electronic prescribing records at Besançon/Montbéliard and local oncology-team identification at Dijon, followed by medical-record review.
6b	**Matching**	For matched studies, give matching criteria and numbers of exposed/unexposed participants.	Not applicable	No matching was used.
7	**Variables**	Clearly define outcomes, exposures, predictors, potential confounders, and diagnostic criteria.	Main text, Sections 2.2–2.4	The MCP regimen, PFS, OS, ORR, DCR, safety outcomes, Mir-comparable subgroup, radiation-associated sarcoma status, and collected clinicopathologic and treatment variables are defined.
8	**Data sources / measurement**	For each variable of interest, describe data sources and measurement/assessment methods.	Main text, Sections 2.1, 2.3 and 2.4	Electronic medical records, local radiological assessment using RECIST 1.1, retrospective CTCAE v5.0 grading, and the clinical/treatment variables collected at each center are described.
9	**Bias**	Describe efforts to address potential sources of bias.	Main text, Sections 2.4 and 4.6–4.7	The same eligibility criteria, outcome definitions, and data-collection variables were applied across all three centers, although case-identification strategies differed by center. Selection bias, confounding by indication, adverse-event under-ascertainment, temporal changes, histologic heterogeneity, missing grade data, and subgroup imprecision are discussed.
10	**Study size**	Explain how the study size was determined.	Main text, Sections 2.1, 2.5 and 3.1	No formal sample-size calculation was performed; study size was determined by the number of eligible patients identified during the predefined study period.
11	**Quantitative variables**	Explain how quantitative variables were handled, including any groupings and their rationale.	Main text, Sections 2.3 and 2.5; Tables 1–2; Supplementary Tables S3–S8	Continuous variables are summarized using medians, IQRs, and ranges, and category boundaries are displayed. The >65 years first-/second-line subgroup is explicitly motivated as the closest available comparison with Mir et al.
12a	**Statistical methods**	Describe all statistical methods, including methods used to control for confounding.	Main text, Section 2.5; Supplementary Tables S3–S8	Kaplan–Meier estimation and Cox proportional-hazards models are reported; the criterion for entry into multivariable modeling is stated and adjusted estimates are presented where applicable.
12b	**Subgroups / interactions**	Describe methods used to examine subgroups and interactions.	Main text, Sections 2.3 and 3.4; Supplementary Tables S5–S8	The Mir-comparable subgroup and exploratory prognostic analyses are reported. No formal interaction testing was performed or reported.
12c	**Missing data**	Explain how missing data were addressed.	Main text, Tables 1–2; Supplementary Tables S3–S8	No imputation was performed. Missing values are reported explicitly; descriptive analyses use available data and regression models use available cases for variables included in each model.
12d	**Loss to follow-up / censoring**	For cohort studies, explain how loss to follow-up and censoring were addressed.	Main text, Section 2.3	PFS censoring at the date of the last evaluable radiological assessment and OS censoring at the date of last follow-up are explicitly reported.
12e	**Sensitivity analyses**	Describe any sensitivity analyses.	Not applicable	No formal sensitivity analysis was performed; subgroup and cross-study analyses were exploratory/comparative rather than sensitivity analyses.
13a	**Participant numbers**	Report numbers of individuals at each stage of the study.	Main text, Section 3.1, Figure 1; Tables 1–2	Sixty-four potentially eligible patients were identified. Four were excluded, and 60 patients were included in the final analysis. The participant selection process is presented in Figure 1. Subgroup denominators are reported in the main text and Tables 1–2.
13b	**Non-participation**	Give reasons for non-participation at each stage.	Main text, Section 3.1, Figure 1	Four of the 64 potentially eligible patients were excluded: two because of insufficient accessible medical records and two because their histological diagnoses fell outside the predefined eligibility criteria. The reasons for exclusion are reported in Section 3.1 and illustrated in Figure 1.
13c	**Flow diagram**	Consider use of a participant flow diagram.	Main text, Section 3.1, Figure 1	A participant flow diagram is provided in Figure 1, showing the identification of 64 potentially eligible patients, the exclusion of four patients with documented reasons, and the final inclusion of 60 patients across the three participating centers.
14a	**Descriptive data**	Give participant characteristics and information on exposures and potential confounders.	Main text, Tables 1–2	Detailed demographic, disease, treatment, prior-therapy, radiotherapy, and MCP exposure characteristics are reported.
14b	**Missing descriptive data**	Indicate the number of participants with missing data for each variable of interest.	Main text, Tables 1–2; Supplementary Tables S3–S8	Unknown/missing counts and available-data denominators are displayed where applicable; regression-table denominators document available-case analyses.
14c	**Follow-up time**	Summarize follow-up time.	Main text, Section 3.2; Supplementary Figure S2	At database lock, the median observed time from MCP initiation to death or last documented follow-up was 6.3 months (IQR, 2.9–11.4 months). Individual patient treatment and follow-up trajectories are additionally presented in Supplementary Figure S2.
15	**Outcome data**	Report numbers of outcome events or summary measures over time.	Main text, Sections 3.2–3.4; Supplementary Tables S1–S2	PFS, OS, ORR, DCR, adverse events, treatment discontinuation, subsequent treatment, and survival-event counts in the regression tables are reported.

Item	Section	STROBE reporting recommendation (paraphrased)	Location in manuscript	Manuscript-specific reporting note
**16a**	**Main results**	Give unadjusted and, where applicable, adjusted estimates with precision; identify variables adjusted for.	Main text, Sections 3.2 and 3.4; Supplementary Tables S3–S8	Survival medians with 95% CIs and Cox HRs with 95% CIs are reported; the multivariable OS model and variables included in that model are shown in Table S4.
**16b**	**Category boundaries**	Report category boundaries when continuous variables are categorized.	Main text, Tables 1–2; Supplementary Tables S3–S8	Age and number of metastatic sites are presented using explicit category boundaries. ECOG performance status, treatment line, and histological grade are reported in their respective categories.
**16c**	**Absolute risk**	When relevant, translate relative-risk estimates into absolute risk over a meaningful period.	Not applicable	Primary outcomes are survival distributions and tumor-response measures rather than relative-risk estimates requiring translation into absolute risk.
**17**	**Other analyses**	Report subgroup, interaction, and other analyses.	Main text, Sections 3.2–3.4 and 4.2–4.4; Supplementary Tables S3–S9	Mir-comparable subgroup analyses, exploratory prognostic analyses, radiation-associated sarcoma subgroup assessment, and a descriptive cross-study comparison are reported.
**18**	**Key results**	Summarize key results with reference to study objectives.	Main text, Section 4.1	The Discussion opens with the principal efficacy and safety findings and explicitly relates them to the external-validation objective.
**19**	**Limitations**	Discuss study limitations, including potential sources and direction/magnitude of bias or imprecision.	Main text, Sections 4.6–4.7	The retrospective design, selection/confounding, adverse-event under-ascertainment, long study period, histologic heterogeneity, missing grade data, small subgroups, regimen differences, and lack of translational analyses are discussed. The likely direction of adverse-event under-ascertainment is specified, and subgroup imprecision is acknowledged.
**20**	**Interpretation**	Give a cautious overall interpretation considering objectives, limitations, multiplicity, and other evidence.	Main text, Sections 4.1–5	Interpretation is cautious, integrates the Mir series and prospective evidence, distinguishes efficacy from tolerability, and avoids claiming equivalence with standard chemotherapy.
**21**	**Generalisability**	Discuss the generalisability/external validity of the results.	Main text, Sections 4.5, 4.7 and 5	The multicenter real-world design supports applicability to routine practice, particularly in older or frail patients, while the Discussion limits extrapolation across histologies, patient fitness, and differing MCP regimens.
**22**	**Funding**	State the source of funding and the role of funders.	Main text, Funding statement	The manuscript states: “This research received no external funding”; therefore, no funder role applies.
