# Supplementary material for: Real-World Activity and Safety of Metronomic Cyclophosphamide in Advanced Soft-Tissue Sarcoma: The Multicenter MeRCY Study
Source: Cancers (Basel). 2026 Oct 30;18(21):3497. doi: 10.3390/cancers18213497 (PMC13761905; doi:10.3390/cancers18213497)
Supplement: Supplementary file 1 [file cancers-18-03497-s001.zip › Supplementary Materials.pdf]

Supplementary Materials

Activity and Safety of Metronomic Cyclophosphamide in Advanced Soft-Tissue Sarcoma: A Multicenter Retrospective External Validation Study

Clément Bolognini et al.

Supplementary figures and tables accompanying the main manuscript are presented below.

Contents

Figure S1. Progression-free survival and overall survival in patients treated with metronomic cyclophosphamide as first- or second-line therapy.
Figure S2. Swimmer plot.
Table S1. Reasons for metronomic cyclophosphamide discontinuation and subsequent systemic treatment.
Table S2. Treatment-related adverse events during metronomic cyclophosphamide therapy.
Table S3. Univariable Cox regression analysis of progression-free survival in the overall population.
Table S4. Univariable and multivariable Cox regression analyses of overall survival in the overall population.
Table S5. Univariable Cox regression analysis of progression-free survival in patients treated with metronomic cyclophosphamide as first- or second-line therapy.
Table S6. Univariable Cox regression analysis of overall survival in patients treated with metronomic cyclophosphamide as first- or second-line therapy.
Table S7. Univariable Cox regression analysis of progression-free survival in patients aged >65 years treated with metronomic cyclophosphamide as first- or second-line therapy.
Table S8. Univariable Cox regression analysis of overall survival in patients aged >65 years treated with metronomic cyclophosphamide as first- or second-line therapy.
Table S9. Comparison of studies evaluating metronomic cyclophosphamide in advanced soft-tissue sarcoma.

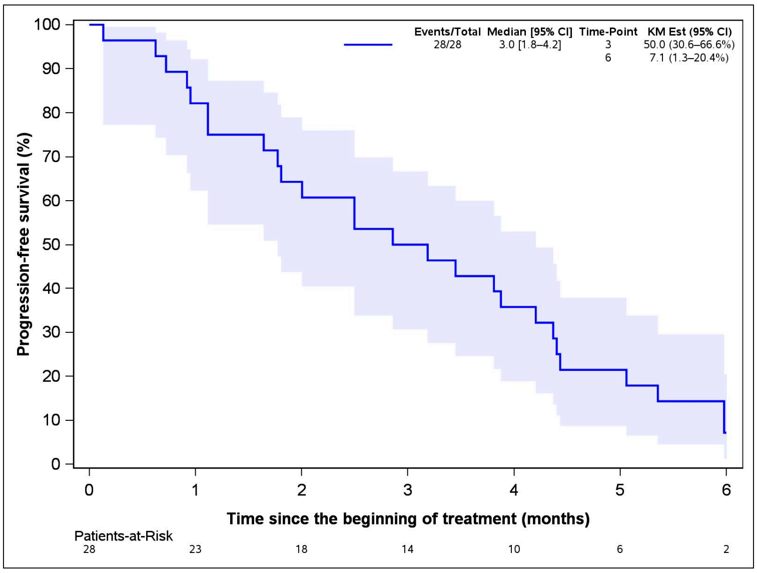

(A)

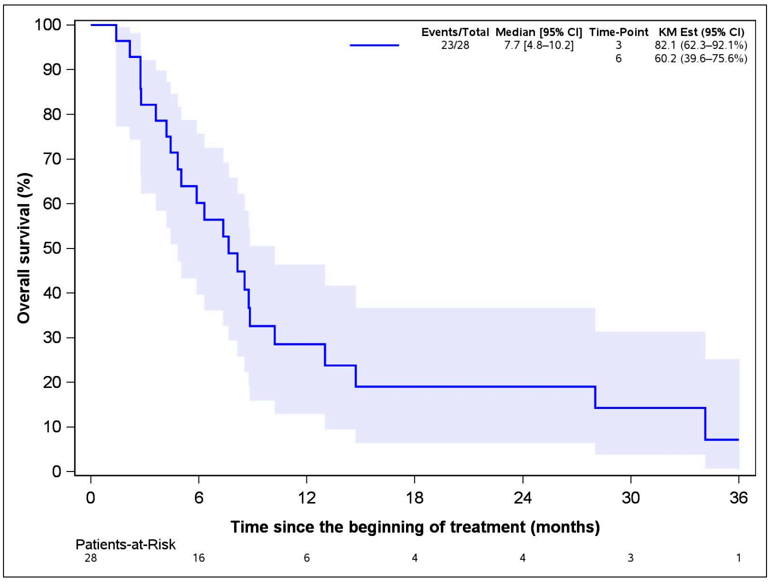

(B)

Figure S1. Progression-free survival and overall survival in patients treated with metronomic cyclophosphamide as first- or second-line therapy. (A) Progression-free survival; (B) overall survival.

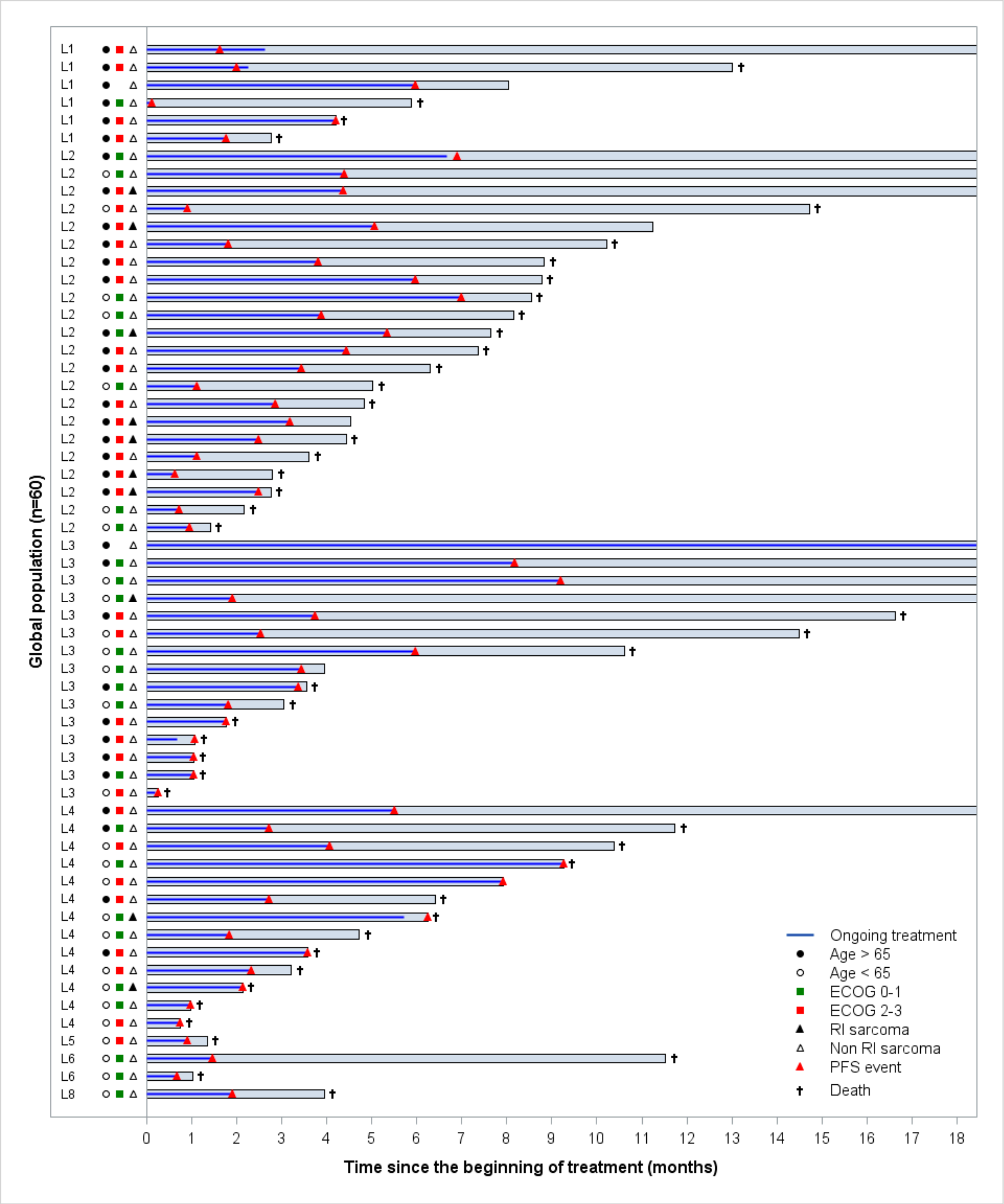

Figure S2. Swimmer plot. Each horizontal bar represents one patient. Symbols indicate age group, ECOG performance status, radiation-induced status, progression-free survival event, and death, as displayed in the figure legend.

Table S1. Reasons for metronomic cyclophosphamide discontinuation and subsequent systemic treatment.

	Total	Line of treatment and age at MCP initiation	
	**Total**	**L1–L2 overall**	**L1–L2 >65 years**
Sample size, No.	60	28	21
**Reason for MCP discontinuation, No. (%)**			
Toxicity	2 (3%)	2 (7%)	2 (10%)
Progressive disease	46 (77%)	25 (89%)	18 (86%)
Death	12 (20%)	1 (4%)	1 (5%)
**Number of subsequent treatment lines, No. (%)**			
0	42 (70%)	20 (71%)	16 (76%)
1	10 (17%)	6 (21%)	5 (24%)
2	6 (10%)	1 (4%)	0 (0%)
3	1 (2%)	0 (0%)	0 (0%)
4	1 (2%)	1 (4%)	0 (0%)
Median (IQR)	0 (0-1)	0 (0-1)	0 (0-0)
Min-max	0-4	0-4	0-1
**Delay from MCP discontinuation until death, Months (range)**			
Median (IQR)	2.2 (0.3-6.3)	2.8 (1.5-5.8)	2.6 (2.0-5.4)
Min-max	0-44.6	0-29.7	0-23.7

Percentages may not total 100 due to rounding. All deaths were related to tumor progression. L1–L2, first- or second-line treatment; IQR, interquartile range; MCP, metronomic cyclophosphamide.

Table S2. Treatment-related adverse events during metronomic cyclophosphamide therapy.

	Total	Line of treatment and age at MCP initiation	
	**Total**	**L1–L2 overall**	**L1–L2 >65 years**
Sample size, No.	60	28	21
**Any grade imputable toxicity reported, No. (%)**			
Yes	16 (27%)	10 (36%)	7 (33%)
No	44 (73%)	18 (64%)	14 (67%)
**Dose adjustment, No. (%)**			
Yes	2 (3%)	1 (4%)	1 (5%)
No	58 (97%)	27 (96%)	20 (95%)
**Etiology of MCP dose adaptation, if any, No. (%)**			
Clinical toxicity	2 (100%)	1 (100%)	1 (100%)
Biological toxicity	0 (0%)	0 (0%)	0 (0%)

L1–L2, first- or second-line treatment; MCP, metronomic cyclophosphamide.

Table S3. Univariable Cox regression analysis of progression-free survival in the overall population.

	Univariable analysis				
	**N**	**events**	**HR**	**95% CI**	**P-value**
**Gender**					
Female	32	32	1		
Male	28	28	1.087	0.651-1.815	0.7507
**Age at MCP initiation**					
<65	27	27	1		
>65	33	33	0.904	0.538-1.519	0.7026
**Stage at diagnosis**					
Unresectable or metastatic at diagnosis	27	27	1		
Metachronous metastases	33	33	0.764	0.454-1.285	0.3098
**ECOG PS**					
0-1	26	26	1		
2-3	32	32	1.538	0.882-2.681	0.1294
Number of metastatic sites					0.6349
0	16	16	1		
1	17	17	1.126	0.556-2.281	0.7413
>1	27	27	1.347	0.713-2.544	0.3584
Grade					0.2056
I	13	13	1		
II	15	15	0.650	0.302-1.399	0.2710
III	19	19	1.221	0.597-2.494	0.5846
**Radiation-induced sarcoma**					
No	50	50	1		
Yes	10	10	1.043	0.523-2.081	0.9051
**Primary tumor surgery**					
No	26	26	1		
Yes	34	34	1.015	0.606-1.701	0.9544
**MCP treatment line**					
L1-L2	28	28	1		
L3+	32	32	0.824	0.482-1.407	0.4780
**Concomitant corticosteroid use**					
No	48	48	1		
Yes	12	12	1.332	0.684-2.595	0.3987

ECOG PS, Eastern Cooperative Oncology Group performance status; MCP, metronomic cyclophosphamide; CI, confidence interval; HR, hazard ratio.

Table S4. Univariable and multivariable Cox regression analyses of overall survival in the overall population.

	Univariable analysis					Multivariable analysis				
	N	events	HR	95% CI	p	N	events	HR	95% CI	p
Gender						46	40			
Female	32	27	1							
Male	28	26	1.240	0.719-2.139	0.4397					
**Age at MCP initiation**										
<65	27	25	1							
>65	33	28	0.696	0.401-1.206	0.1959					
**Stage at diagnosis**										
Unresectable or metastatic at diagnosis	27	22	1							
Metachronous metastases	33	31	0.686	0.391-1.202	0.1878					
**ECOG PS**										
0-1	26	24	1							
2-3	32	28	1.035	0.598-1.789	0.9027					
Number of metastatic sites					0.6795					
0	16	13	1							
1	17	15	0.818	0.385-1.734	0.5997					
>1	27	25	1.091	0.553-2.154	0.8011					
Grade					0.0300					0.0706
I	13	9	1					1		
II	15	14	1.829	0.761-4.393	0.1769			1.730	0.702-4.262	0.2335
III	19	17	3.221	1.336-7.765	0.0092			2.921	1.152-7.558	0.0241
**Radiation-induced sarcoma**										
No	50	45	1							
Yes	10	8	0.966	0.453-2.060	0.9284					
**Primary tumor surgery**										
No	26	23	1					1		
Yes	34	30	0.574	0.320-1.031	0.0631			0.665	0.331-1.338	0.2529
**MCP treatment line**										
L1-L2	28	23	1							
L3+	32	30	1.349	0.781-2.333	0.2830					
**Concomitant corticosteroid use**										
No	48	41	1					1		
Yes	12	12	1.735	0.901-3.341	0.0995			1.079	0.479-2.432	0.8540

ECOG PS, Eastern Cooperative Oncology Group performance status; MCP, metronomic cyclophosphamide; CI, confidence interval; HR, hazard ratio.

Table S5. Univariable Cox regression analysis of progression-free survival in patients treated with metronomic cyclophosphamide as first- or second-line therapy.

	**Univariable analysis**				
	**N**	**events**	**HR**	**95% CI**	**p**
**Gender**					
Female	14	14	1		
Male	14	14	0.876	0.409-1.876	0.7331
**Age at MCP initiation**					
<65	7	7	1		
>65	21	21	0.994	0.396-2.498	0.9902
**Stage at diagnosis**					
Unresectable or metastatic at diagnosis	15	15	1		
Metachronous metastases	13	13	1.229	0.573-2.638	0.5960
**ECOG PS**					
0-1	9	9	1		
2-3	18	18	1.781	0.719-4.411	0.2124
Number of metastatic sites					0.6736
0	11	11	1		
1	9	9	0.695	0.267-1.807	0.4550
>1	8	8	1.076	0.425-2.722	0.8778
Grade					0.8502
I	5	5	1		
II	7	7	1.074	0.303-3.809	0.9118
III	11	11	1.331	0.419-4.226	0.6275
**Radiation-induced sarcoma**					
No	21	21	1		
Yes	7	7	1.070	0.442-2.588	0.8809
**Primary tumor surgery**					
No	14	14	1		
Yes	14	14	1.089	0.507-2.336	0.8270
**Concomitant corticosteroid use**					
No	22	22	1		
Yes	6	6	2.363	0.904-6.177	0.0793

No multivariable model was fitted because fewer than two variables met the $p<0.10$ screening threshold. ECOG PS, Eastern Cooperative Oncology Group performance status; MCP, metronomic cyclophosphamide; CI, confidence interval; HR, hazard ratio.

Table S6. Univariable Cox regression analysis of overall survival in patients treated with metronomic cyclophosphamide as first- or second-line therapy.

	**Univariable analysis**				
	**N**	**events**	**HR**	**95% CI**	**p**
**Gender**					
Female	14	11	1		
Male	14	12	0.977	0.429-2.221	0.9549
**Age at MCP initiation**					
<65	7	7	1		
>65	21	21	0.820	0.334-2.017	0.6658
**Stage at diagnosis**					
Unresectable or metastatic at diagnosis	15	11	1		
Metachronous metastases	13	12	0.804	0.343-1.885	0.6154
**ECOG PS**					
0-1	9	8	1		
2-3	18	15	1.061	0.429-2.625	0.8978
Number of metastatic sites					0.2269
0	11	8	1		
1	9	8	0.437	0.152-1.259	0.1254
>1	8	7	1.036	0.368-2.911	0.9472
Grade					0.1423
I	5	5	1		
II	7	2	3.517	0.718-17.227	0.1208
III	11	7	4.862	1.012-23.361	0.0483
**Radiation-induced sarcoma**					
No	21	18	1		
Yes	7	5	1.080	0.397-2.938	0.8804
**Primary tumor surgery**					
No	14	12	1		
Yes	14	11	0.420	0.168-1.050	0.0636
**Concomitant corticosteroid use**					
No	22	17	1		
Yes	6	6	1.896	0.736-4.883	0.1849

No multivariable model was fitted because fewer than two variables met the $p<0.10$ screening threshold. ECOG PS, Eastern Cooperative Oncology Group performance status; MCP, metronomic cyclophosphamide; CI, confidence interval; HR, hazard ratio.

Table S7. Univariable Cox regression analysis of progression-free survival in patients aged >65 years treated with metronomic cyclophosphamide as first- or second-line therapy.

	Univariable analysis				
	N	events	HR	95% CI	p
**Gender**					
Female	13	13	1		
Male	8	8	1.019	0.411-2.524	0.9678
**Stage at diagnosis**					
Unresectable or metastatic at diagnosis	11	11	1		
Metachronous metastases	10	10	1.104	0.454-2.684	0.8268
**ECOG PS**					
0-1	3	3	1		
2-3	17	17	3.217	0.695-14.888	0.1349
Number of metastatic sites					0.9617
0	9	9	1		
1	6	6	1.062	0.352-3.205	0.9147
>1	6	6	1.163	0.403-3.359	0.7801
Grade					0.7450
I	4	4	1		
II	5	5	1.488	0.342-6.467	0.5962
III	10	10	1.665	0.453-6.123	0.4429
**Radiation-induced sarcoma**					
No	14	14	1		
Yes	7	7	1.157	0.447-2.998	0.7634
**Primary tumor surgery**					
No	10	10	1		
Yes	11	11	0.786	0.317-1.948	0.6028
**Concomitant corticosteroid use**					
No	17	17	1		
Yes	4	4	2.622	0.828-8.303	0.1012

No multivariable model was fitted because fewer than two variables met the p<0.10 screening threshold. ECOG PS, Eastern Cooperative Oncology Group performance status; MCP, metronomic cyclophosphamide; CI, confidence interval; HR, hazard ratio.

Table S8. Univariable Cox regression analysis of overall survival in patients aged >65 years treated with metronomic cyclophosphamide as first- or second-line therapy.

	Univariable analysis				
	N	events	HR	95% CI	p
**Gender**					
Female	13	10	1		
Male	8	6	1.020	0.369-2.819	0.9701
**Stage at diagnosis**					
Unresectable or metastatic at diagnosis	11	7	1		
Metachronous metastases	10	9	1.410	0.521-3.817	0.4992
**ECOG PS**					
0-1	3	2	1		
2-3	17	14	1.723	0.388-7.646	0.4743
Number of metastatic sites					0.6875
0	9	6	1		
1	6	5	0.706	0.204-2.445	0.5826
>1	6	5	1.261	0.377-4.223	0.7068
Grade					0.2294
I	4	1	1		
II	5	5	6.599	0.717-60.731	0.0957
III	10	8	5.866	0.712-48.345	0.1002
**Radiation-induced sarcoma**					
No	14	11	1		
Yes	7	5	1.135	0.393-3.280	0.8152
**Primary tumor surgery**					
No	10	8	1		
Yes	11	8	0.514	0.185-1.431	0.2026
**Concomitant corticosteroid use**					
No	17	12	1		
Yes	4	4	1.652	0.522-5.221	0.3929

No multivariable model was fitted because fewer than two variables met the p<0.10 screening threshold. ECOG PS, Eastern Cooperative Oncology Group performance status; MCP, metronomic cyclophosphamide; CI, confidence interval; HR, hazard ratio.

Table S9. Comparison of studies evaluating metronomic cyclophosphamide in advanced soft-tissue sarcoma.

**Characteristic**	**Mir et al. 2011 [25]**	**Quek et al. 2017 [26]**	**Present study (MeRCY comparable subgroup)**	**METROPHOLYS 2025 [27]**
Design	Retrospective single-center series	Prospective single-arm phase II	Multicenter retrospective cohort; comparable subgroup	Randomized multicenter phase II
MCP-treated patients, n	26	23	21	21
Population / treatment line	Age ≥65 years; 1L (n=19) or 2L (n=7)	Advanced sarcoma; generally after failure of 1L chemotherapy; older patients could be treatment-naïve	Age >65 years; 1L (n=6) or 2L (n=15)	Age ≥70 years; 1L
Median age, years	72	61	80	75
MCP regimen	Cyclophosphamide 100 mg twice daily + prednisolone 20 mg/day during week 1 of each 2-week cycle	Cyclophosphamide 50 mg once daily (21-day cycles) until progression/intolerance	Cyclophosphamide 50 mg twice daily, 1 week on / 1 week off; corticosteroids in 4/21 (19%)	Cyclophosphamide 50 mg once daily until progression/intolerable toxicity
ORR	26.9%	0%	10%	6.7%
DCR / 12-week clinical benefit	69.2%	17%	29%	NR
Median PFS, months	6.8	1.3	3.2	2.3
Median OS, months	NR	NR	7.7	20.9
Selected safety findings	Grade ≥3 lymphopenia 81%; grade 3 anemia 8%; grade 3 thrombocytopenia 8%	Two treatment-related grade 3 events	No grade 3–4 treatment-related adverse events	Any-grade adverse events 66.7%; 4 serious adverse events

Abbreviations: 1L, first-line treatment; 2L, second-line treatment; DCR, disease control rate; MCP, metronomic cyclophosphamide; NR, not reported; ORR, objective response rate; OS, overall survival; PFS, progression-free survival; STS, soft-tissue sarcoma. Cross-study comparisons are descriptive because populations, dosing regimens, and study designs differ.
